# Supplementary figures and images for: Collagen polarization promotes epithelial elongation by stimulating locoregional cell proliferation
Source: eLife. 2021 Oct 18;10:e67915. doi: 10.7554/eLife.67915 (PMC8550756; doi:10.7554/eLife.67915)

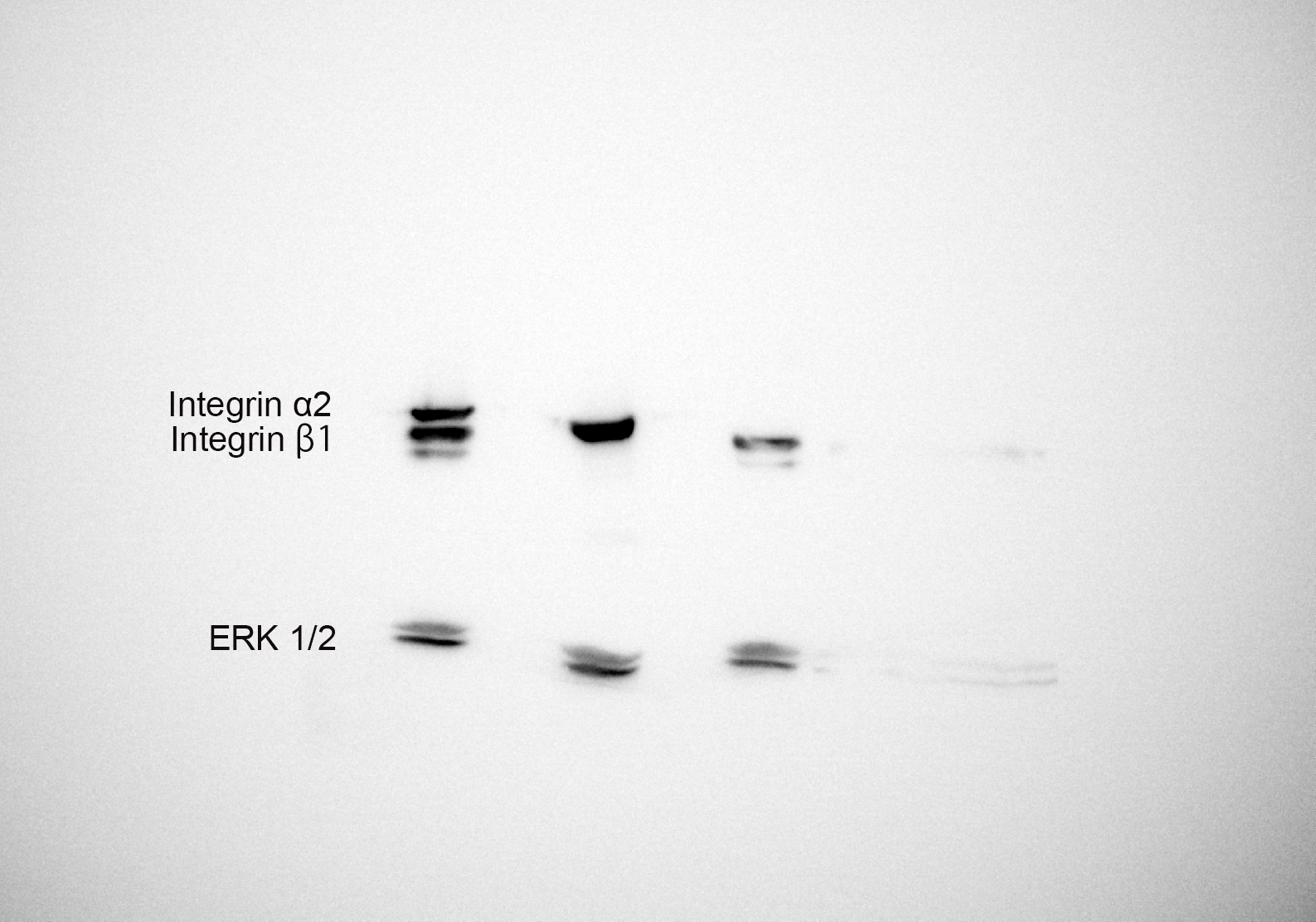

Supplement: Figure 7—source data 2. [file elife-67915-fig7-data2.zip › Figure 7-Source data 2.tif]
